# Findings and Implications of the Global Burden of Disease 2010 Study for the Pacific Islands

**DOI:** 10.5888/pcd11.130344

**Published:** 2014-05-08

**Authors:** Damian Hoy, Adam Roth, Kerri Viney, Yvan Souares, Alan D. Lopez

**Affiliations:** Author Affiliations: Adam Roth, Kerri Viney, Yvan Souares, Alan D. Lopez, Secretariat of the Pacific Community, Noumea, New Caledonia.

## Abstract

The Global Burden of Disease 2010 Study is the largest study of its kind. It provides a large volume of information about the global burden of disease and associated risk factors. It estimates that lower respiratory infections, diabetes, diarrhea, and tuberculosis cause the greatest burden in the Pacific, and noncommunicable diseases caused a substantially greater burden in 2010 compared with 1990. Although the Pacific is considered to be a region rich in data, very little of these data has been analyzed, synthesized, and made publically available. Consequently, burden estimates for the Pacific are derived from models built with very limited data, and it is difficult to know how accurate they are. Health information in the Pacific needs strengthening, particularly in relation to data collection, analysis, use, and sharing. This will improve the reliability and comparability of burden of disease estimates.

## Introduction

Burden of disease (BoD) research is likely to be a useful tool for policy and programming in the Pacific, as elsewhere. Progressive assessments of the global BoD, which began in the early 1990s, have represented a major step in the ability of governments and researchers to summarize a population’s health. BoD research takes both fatal and nonfatal health outcomes into account and is, therefore, a far more comprehensive measurement framework for assessing disease burden than simply relying on mortality alone (1,2). Results of BoD research have been used throughout the world by both government and nongovernment agencies in assessing health priorities, informing the allocation of resources for health, and evaluating the potential costs and benefits of public health interventions (2,3).

BoD studies describe the burden arising from specific diseases and injuries or that is attributable to specific risk factors, by using a summary measure called disability-adjusted life years (DALYs). This quantifies loss of healthy years of life attributable to premature death and illness against an ideal that everyone in the population lives into old age free of disease (4). DALYs are calculated by adding years of life lost in a population attributable to premature mortality (YLLs) to healthy years of life lost in a population attributable to disability (YLDs). This means that diseases can be compared across populations and within populations over time (2). One of the philosophies of BoD research is to endeavor to incorporate all conditions of public health importance, not just those with available and accurate data. Having said that, studies included in global BoD assessments are usually assessed for quality or risk of bias, and those considered to be poor quality or at high risk of bias are generally excluded. BoD research also measures and attributes disease and injury burden to major risk factors and has been used as the basis on which to compare the cost-effectiveness of population health interventions (2,5).

The most recent Global Burden of Disease Study (GBD 2010) took place from 2007 to 2012, and was a collaboration between numerous universities and experts in epidemiology and other areas of public health research from around the world. Disease burden was calculated for 291 causes in the 21 GBD world regions for 1990, 2005, and 2010 (5). GBD 2010 culminated in 5 key articles (5–9). We discuss and contextualize the findings of GBD 2010 for Pacific Island countries and territories (PICTs), as represented by the Oceania region, and suggest ways of improving the reliability and comparability of national health data through strengthening health information systems. This will have profound benefits to local communities, including improving the reliability and comparability of BoD estimates.

## Background

Life expectancies in PICTs are generally low (10), and, in many countries, these have not seen significant improvement over the past 20 years (11). Many PICTs continue to have a high incidence of communicable diseases (11,12) and are experiencing increasing prevalences of noncommunicable diseases. This double BoD has increased over recent years and is having a profound effect on life expectancy and well-being (11).

The region also faces challenges with widely dispersed populations, limited resources, and fragmented health systems. Health systems are highly reliant on funding from development partners, and are thus heavily influenced by regional and global health priorities. Reliable data on the comparative impact of specific diseases and risk factors are critical for regional and national health planning and evaluation (2). Although the Pacific is considered to be a region rich in data (13–16), very little of these data has been analyzed, synthesized, and made publicly available.

Oceania was one of the 21 regions assessed in GBD 2010. It comprised the following PICTs (Table 1): American Samoa, Cook Islands, Fiji, French Polynesia, Guam, Kiribati, Marshall Islands, Federated States of Micronesia, Nauru, New Caledonia, Niue, Norfolk Island, Northern Mariana Islands, Palau, Papua New Guinea, Pitcairn Islands, Samoa, Solomon Islands, Tokelau, Tonga, Tuvalu, Vanuatu, and Wallis and Futuna. This group of 23 countries and territories includes all 22 of the Secretariat of the Pacific Community member countries and territories. Norfolk Island is not a member of the Secretariat of the Pacific Community; it has a population of 2,302, and therefore has minimal impact on the overall results for Oceania (18).

**Table 1:** Population of Pacific Island Countries and Territories Included in Global Burden of Diseases Study, 2010 (17)

Country or Territory	Population (%)
**Total**	**9,829,322** (100)
American Samoa	65,896 (0.7)
Cook Islands	15,529 (0.2)
Fiji	847,793 (8.6)
French Polynesia	268,767 (2.7)
Guam	187,140 (1.9)
Kiribati	100,835 (1.0)
Marshall Islands	54,439 (0.6)
Federated States of Micronesia	102,959 (1.0)
Nauru	9,976 (0.1)
New Caledonia	248,995 (2.5)
Niue	1,479 (0.0)
Northern Mariana Islands	63,072 (0.6)
Palau	20,518 (0.2)
Papua New Guinea	6,744,955 (68.6)
Pitcairn Islands	66 (0.0)
Samoa	183,123 (1.9)
Solomon Islands	539,469 (5.5)
Tokelau	1,165 (0.0)
Tonga	103,365 (1.1)
Tuvalu	11,149 (0.1)
Vanuatu	245,376 (2.5)
Wallis and Futuna	13,256 (0.1)

## Summary of Findings

The overall BoD in the PICTs was estimated to increase 20% from 4.0 million DALYs in 1990 (uncertainty interval [UI], 3.5 million to 4.6 million) to 4.8 million DALYs in 2010 (UI, 3.9 million to 5.8 million). However, when population increase was taken into account by calculating the DALY rate per 1,000 population, the BoD actually decreased by 23%, from 621 DALYs per 1,000 population in 1990 (UI, 546 to 714) to 481 DALYs per 1,000 population in 2010 (UI, 393 to 586) (5). This suggests an improved average health status per capita, although this was not significant at the .05 level. Table 2 shows the top 10 rankings for DALYs, YLLs, YLDs, and risk factors in PICTs for 1990 and 2010.

**Table 2:** Top 10 Rankings for Disability-Adjusted Life Years (DALYs), Years of Life Lost in a Population Attributable to Premature Mortality (YLLs), Years of Life Lost in a Population Attributable to Disability (YLDs), and Risk Factors for Disease and Injury in the Pacific Island Countries and Territories, 1990 and 2010

Rank	1990				2010			
	DALYs (5)	YLLs (6)	YLDs (7)	Risk Factors (9)	DALYs (5)	YLLs (6)	YLDs (7)	Risk Factors (9)
1	Lower respiratory infections	Lower respiratory infections	Iron-deficiency anemia	Household air pollution	Lower respiratory infections	Lower respiratory infections	Major depressive disorder	High fasting plasma glucose
2	Diarrheal diseases	Diarrheal diseases	Major depressive disorder	Childhood underweight	Diabetes	Diabetes	Low back pain	High body mass index
3	Malaria	Malaria	Low back pain	Tobacco smoking	Diarrheal diseases	Diarrheal diseases	Iron-deficiency anemia	Tobacco smoking
4	Tuberculosis	Protein energy malnutrition	Hookworm disease	Suboptimal breastfeeding	Tuberculosis	Malaria	Diabetes	Household air pollution
5	Protein energy malnutrition	Preterm birth complications	Tuberculosis	High fasting plasma glucose	Malaria	Ischemic heart disease	Hookworm disease	Alcohol use
6	Preterm birth complications	Tuberculosis	Anxiety disorders	Alcohol use	Ischemic heart disease	Tuberculosis	Tuberculosis	High blood pressure
7	Meningitis	Meningitis	Chronic obstructive pulmonary disease	High blood pressure	Preterm birth complications	Preterm birth complications	Anxiety disorders	Physical inactivity
8	Diabetes	Poisonings	Diabetes	High body mass index	Asthma	Cerebrovascular disease	Neck pain	Childhood underweight
9	Poisonings	Diabetes	Neck pain	Iron deficiency	HIV/AIDS	Meningitis	Chronic obstructive pulmonary disease	Diet low in fruits
10	Ischemic heart disease	Ischemic heart disease	Falls	Diet low in fruits	Meningitis	HIV/AIDS	Other musculoskeletal disorders	Suboptimal breastfeeding

The GBD 2010 estimates of deaths by age and sex were derived for 187 countries, using several methods and a large database of vital registration, verbal autopsy, surveillance, and other sources (8). Deaths by age and sex attributable to specific diseases were modeled using the Cause of Death Ensemble Modeling strategy (6). The crude death rate for PICTs was estimated to be 10.3 per 1000 in 1990 (UI, 8.0 to 14.1) and 9.0 per 1000 in 2010 (UI, 6.6 to 12.8) (8). Life expectancy at birth for the PICTs in 1990 was estimated to be 56 years (UI, 51 to 61); this was 55 years for males (UI, 46 to 61) and 58 years for females (UI, 51 to 64). By 2010, it was estimated that this had increased to 60 years (UI, 55 to 65); 59 years for males (UI, 51 to 66) and 62 years for females (UI, 55 to 69) (19). The overall BoD from premature mortality was estimated to be 3.3 million YLLs in 1990 and this increased to 3.7 million YLLs in 2010. YLLs were responsible for 81% of the overall disease burden in 1990, decreasing slightly to 77% in 2010 (20).

YLDs were estimated for 1,160 sequelae of the list of diseases and injuries. They were calculated by multiplying the prevalence of the sequelae by a disability weight for the sequelae. The disability weight reflects the level of severity of each health state on a scale of 0 to 1. Prevalence was most commonly derived through systematic reviews and meta-regression. The proportion of the overall burden attributable to YLDs is approximately 23% in the PICTs and has increased slightly since 1990 (5).

The GBD 2010 also included a component on comparative risk assessment, which involved estimating disease burden arising from specific risk factors. To do this, population-attributable fractions were determined on the basis of estimates of exposure prevalence in the population by age and sex and relative risk arising from that exposure (based on the epidemiological literature). High fasting plasma glucose, high body mass index, tobacco smoking, household air pollution, and alcohol use were estimated to be the risk factors causing the greatest burden in the PICTs in 2010 (Table 2).

## Potential Limitations

Despite the clear benefits of GBD 2010, like any research, there are limitations (21); it is important to consider these when interpreting the results. A central GBD philosophy is that some estimate is always better than no estimate. With this in mind, and as BoD research endeavors to incorporate all conditions of public health importance, it is often necessary to predict estimates using models, which are built up from various covariates (6,7,21). Although this ensures an estimate for all causes within a region, these modeled estimates have limitations (22). In PICTs, the greatest limitation is the paucity of data that have been analyzed, synthesized, and made publically available. Consequently, GBD estimates are based largely on these models, and therefore it is difficult to know with confidence how correct the PICTs burden estimates are. A primary challenge for health policy in PICTs is to be able to gain a more confident understanding of the BoD. Improving the availability and quality of data in PICTs will reduce uncertainty around the estimates and consequently improve confidence in their use for policy discussions.

An additional consideration is that the GBD 2010 estimates for PICTs were heavily influenced by the health and demographic profile of Papua New Guinea, as its population constitutes 69% of the total Pacific Islands population (23). Given the health, demographic, and environmental diversity in the region and the pace at which this is changing (11), it is unlikely that the epidemiological profile in Papua New Guinea adequately reflects the region-wide profile. For example, Papua New Guinea has the highest maternal mortality rate in the Pacific, the highest prevalence of HIV infection, and the third highest prevalence of tuberculosis; however, it also has one of the lowest prevalences of diabetes and the lowest prevalence of obesity (10), conditions which are highly prevalent elsewhere in the region. Certain conditions such as dengue fever, chikungunya, cervical cancer, and ciguatera are likely to be underreported and, consequently, their burden is likely to be underestimated (24).

In BoD research, the definition of disability is health loss resulting from episodes of disease and injury, often resulting in impairments of body structures and functions as well as more complex human operations (eg, mobility). Broader constructs of the magnitude of diseases such as participation restriction (a problem experienced by an individual with involvement in life situations), well-being, caretaker burden, increased pressure on health care systems, and economic costs are not included; it is prudent to also consider these broader constructs when examining the effect of disease on populations.

## Improving Reliability and Comparability of Burden Estimates

Improving the reliability, analysis, use, and availability of national-level empirical data is needed to better understand the effect of disease, injury, and common risk factors on PICT populations as well as their progress toward key development indicators such as the Millennium Development Goals (25). This includes information collected through censuses, surveys, civil registration, routine surveillance of diseases and risk factors, and research studies. Table 3 highlights some data improvement needs and potential opportunities to address these. In particular, better data are necessary for those conditions that are estimated to cause a substantial burden but for which there are very little data available for PICTs. These include lower respiratory infections and diarrheal diseases for YLLs and depression and low back pain for YLDs. Common risk factors should continue to be measured periodically, and risk factors that are not well measured, such as household air pollution, should be better studied to determine the extent of population exposure to them and feasible solutions for risk reduction. To achieve this, strategic leadership is needed to ensure that the limited resources for strengthening data collection, analysis, and use in the region are allocated to appropriately monitor leading causes of health loss, promote harmonization of data, and remove duplication of efforts.

**Table 3:** Health Data Improvement Needs and Potential Opportunities in Pacific Island Countries and Territories

Data Needs	Data Already Available	Limitations of These Data	Current Work and Opportunities for Addressing These Limitations
Civil registration and vital statistics (CRVS)	Some data are available.	Coverage and completeness need strengthening to improve the validity, reliability, and comparability of these data.	The Brisbane Accord Group (BAG) has a comprehensive and coordinated plan for strengthening mortality information in Pacific Island countries and territories.
Routinely collected information on the incidence of disease	Most countries have complex facility registers, reporting forms, and medical record rooms.	Improvement is needed to ensure better consistency in data collection, definitions, and diagnosis. Data are often not collated, analyzed, interpreted, and reported on.	Pacific Public Health Surveillance Network (PPHSN) regional partners are undertaking data for decision-making (DDM) training in the Pacific. The Pacific Health Information Network is also working to strengthen country health information systems.
Prevalence of chronic diseases and risk factors (eg, low back pain, depression)	Surveys that are routinely conducted in the Pacific include WHO STEPwise Approach to Surveillance and the Demographic and Health Survey. They provide some chronic disease prevalence data such as prevalence of diabetes and noncommunicable disease risk factors.	Key conditions such as low back pain, depression, anxiety, and neck pain are not included in these surveys.	These surveys need to continue to gather important information on chronic diseases and risk factors. Standardised questions exist for measuring the prevalence of many chronic diseases (eg, low back pain, depression) in population surveys. Surveys such as WHO-STEPs and the Demographic and Health Survey provide opportunities for collecting these data without burdening communities with additional surveys.
Data analysis and usage skills in Pacific Island countries and territories need strengthening to ensure that the data that gets collected gets used	Not applicable.	Not applicable.	PPHSN regional partners are undertaking DDM training in Pacific Island countries and territories. In addition, BAG is conducting training to improve analysis of CRVS data.

A health information system is defined as “an integrated effort to collect, process, report and use health information and knowledge to influence policy-making, programme action, and research” (p. 116) (26). Reliable and timely health information is one of the cornerstones of effective health systems and is critical for health program planning, monitoring and evaluation, prioritization, and accountability to donors and constituencies (27). Several initiatives exist to strengthen health information for PICTs, including the completeness, timeliness, validity, reliability, and comparability of data. There is a critical need for better harmonization to ensure a comprehensive, collective, and integrated approach.

Pacific health ministers are well aware of the issue of the existence of data but the lack of use of these data. They have recommended “development of comprehensive training programmes to develop core competencies in ‘data techs’, ‘epi techs’ and epidemiologists” (p. 46) (28). These data techs and epi techs are not full-blown epidemiologists, but rather people who have some training in data management, analysis, and interpretation. In response, the Pacific Public Health Surveillance Network (PPHSN) regional partners, under the leadership of Secretariat of the Pacific Community, have revitalized the existing PPHSN and Fiji National University collaboration for data for decision-making and are exploring ways to broaden this program to strengthen national and regional capacity for essential public health functions and services in the PICTs.

Concurrently, The Brisbane Accord Group is a collaboration that aims to provide strategic and technical support to countries to strengthen vital statistics systems, providing a more coordinated response from partner agencies’ efforts to achieve this. The overarching aim of the plan is to assist PICTs to improve their routinely collected statistics on birth, deaths, and cause of death, thus providing decision-makers with critical evidence needed for effective planning (29). The Pacific Health Information Network, which was created to provide a mechanism for networking, support, information sharing, and training for people working as health information professionals in the region, could also play a more important role in regional strategies to strengthen country health information systems to reduce uncertainty in BoD estimates.

Operational research is the search for knowledge that informs policy and practice in the short and intermediate term, with a special focus on interventions, strategies, or tools that can enhance the performance (quality, effectiveness, or coverage) of the program in which the research is being conducted (30). Establishing a joint health information and operational research agenda for PICTs, including BoD research in which countries identify common priorities and data and knowledge gaps to guide partners, would facilitate the evidence base for a more informed implementation of policies. Regional and national leadership is needed to enhance the perceived value and use of data.

There are also initiatives to strengthen operational research in PICTs. The Secretariat of the Pacific Community runs an operational research course in collaboration with the International Union Against Tuberculosis and Lung Disease (The Union) and Fiji National University. The course is based on The Union/Médecins Sans Frontières model of operational research training and aims to build the capacity of national health program staff to plan, conduct, and publish operational research and to influence policy and practice with their results. Several other partners such as the Centre for the Prevention of Obesity and Non-communicable Diseases and the School of Public Health and Community Medicine at University of New South Wales have ongoing programs in operational research to strengthen and use noncommunicable disease morbidity and mortality data in the Pacific Islands.

All of the above initiatives have great potential to contribute to rapidly strengthening, rationalizing, and harmonizing existing data collection and analysis efforts, including BoD estimates, to ensure good quality data are available for informing policy and programs in the region. Through those mechanisms mentioned above, regional leadership and effective regional coordination and partnerships are needed, which will provide countries, donors, and other development partners with a strategic and realistic option to collectively and rapidly reduce the lack of knowledge around population health and BoD in the region and how this burden is changing.

## Conclusion

The GBD 2010 estimates have substantial relevance for governments and development partners, among others, when assessing health priorities, allocating resources, and evaluating the potential costs and benefits of public health interventions. A major consideration when interpreting GBD 2010 estimates for PICTs is that estimates are largely the result of models built up from covariates based on limited data. Also, the burden is heavily influenced by the health and demographic profile in Papua New Guinea, which is unlikely to represent the epidemiological situation across all PICTs because of diverse health and demographic profiles. BoD results should be supplemented with information on participation restriction, well-being, caretaker burden, increased pressure on health care systems, and economic costs when examining the effect of disease on populations. Health information in PICTs needs strengthening, particularly in relation to data collection, analysis, use, and sharing. This strengthening should be done in a comprehensive, collective, and integrated way. This will improve the reliability and comparability of data summarizing the population’s health through methods such as BoD.
